# The Effect of Electronic Cigarette User Modifications and E-liquid Adulteration on the Particle Size Profile of an Aerosolized Product

**DOI:** 10.1038/s41598-019-46387-2

**Published:** 2019-07-15

**Authors:** Haley A. Mulder, Jesse L. Patterson, Matthew S. Halquist, Leon Kosmider, Joseph B. McGee Turner, Justin L Poklis, Alphonse Poklis, Michelle R. Peace

**Affiliations:** 1 Department of Forensic Science, Virginia Commonwealth University, Richmond, USA; 2 Department of Pharmaceutics, Virginia Commonwealth University, Richmond, USA; 3 Department of Chemistry, Virginia Commonwealth University, Richmond, USA; 4 Department of Pharmacology & Toxicology, Virginia Commonwealth University, Richmond, USA; 5 Department of Pathology, Virginia Commonwealth University, Richmond, USA

**Keywords:** Drug delivery, Pharmacokinetics, Respiration, Characterization and analytical techniques

## Abstract

Electronic cigarettes (e-cigarettes) are an alternate nicotine delivery system that generate a condensation aerosol to be inhaled by the user. The size of the droplets formed in the aerosol can vary and contributes to drug deposition and ultimate bioavailability in the lung. The growing popularity of e-cigarette products has caused an increase in internet sources promoting the use of drugs other than nicotine (DOTNs) in e-cigarettes. The purpose of this study was to determine the effect of various e-cigarette and e-liquid modifications, such as coil resistance, battery voltage, and glycol and drug formulation, on the aerosol particle size. E-liquids containing 12 mg/mL nicotine prepared in glycol compositions of 100% propylene glycol (PG), 100% vegetable glycerin (VG), or 50:50 PG:VG were aerosolized at three voltages and three coil resistances. Methamphetamine and methadone e-liquids were prepared at 60 mg/mL in 50:50 PG:VG and all e-liquids were aerosolized onto a 10 stage Micro-Orifice Uniform Deposit Impactor. Glycol deposition correlated with drug deposition, and the majority of particles centered between 0.172–0.5 μm in diameter, representing pulmonary deposition. The 100% PG e-liquid produced the largest aerosol particles and the 100% VG and 50:50 PG:VG e-liquids produced ultra-fine particles <0.3 μm. The presence of ultrafine particles indicates that drugs can be aerosolized and reach the pulmonary alveolar regions, highlighting a potential for abuse and risk of overdose with DOTNs aerosolized in an e-cigarette system.

## Introduction

Electronic cigarettes (e-cigarettes) are an electronic nicotine delivery system (ENDS) that contain nicotine formulations to produce an aerosol the user inhales^1,2^. The aerosol from the e-cigarette is created when the battery power supply heats a metal coil housed in the atomizer, which contains a wicking material saturated with a liquid formulation (e-liquid). When the coil heats up, the e-liquid in contact with the coil vaporizes, quickly condenses, and is then delivered to the user when they inhale on the device^2^. The e-liquid typically contains a glycol base mixture of propylene glycol (PG) and vegetable glycerin (VG), the nicotine, and flavorants^1^.

Early generation e-cigarettes were modelled to look like traditional cigarettes and contained a single, non-refillable tank^3^. A currently popular e-cigarette is the rebuildable atomizer (RBA), which has a customizable battery and atomizer system, allowing the user to vary battery voltage, the coil, and wicking configurations within the device^1^. These modifications can be purchased or created by the user. Tank e-cig systems enable users to vary e-liquid formulations according to their preferences, whether they are made by the end user or purchased pre-made. These variations may affect the aerosolization of the active ingredients and, thereby, their absorption from the lung tissue into the bloodstream.

Inhalation of drugs has been demonstrated to be a fast and efficient method for delivering drugs to the pulmonary and systemic regions^4^. Typically, compounds that have aerosol particles with a mean mass aerodynamic diameter (MMAD) less than 5 μm in size are readily deposited into the blood stream via the lung^5,6^. Cigarette smoke has been reported to have a MMAD distribution from 0.3–0.5 μm and experiments assessing nicotine in electronic cigarette aerosol have determined that the MMAD range is from 0.25–0.45 μm^2,7^. Previous studies using 100% propylene glycol formulations generated particle sizes with MMAD less than 1 µm for drugs such as butalbital, buprenorphine, morphine, and scopolamine^8–10^.

The emergence of customizable devices has created a subculture where users can manipulate the devices or the e-liquids to vape drugs other than nicotine (DOTNs). Many of these e-liquids are made by the user, but commercial e-liquids containing illicit and controlled substances, as well as uncontrolled DOTNs, can be found online despite efforts to regulate e-cigarettes and e-liquids^11–15^. Discussions in online forums have outlined ways to infuse the DOTNs into e-liquids, with the most popular drugs of choice being traditionally smoked drugs such as methamphetamine, tetrahydrocannabinol, and heroin^16–18^. The characterization of the aerosols generated with nicotine and DOTNs such as methamphetamine and methadone demonstrate the effectiveness of aerosol drug delivery to the lungs. Demonstrating potential drug delivery of these drugs increases the understanding of their abuse potential.

The purpose of this study was to evaluate change in particle size distribution of the condensation aerosol related to battery power and resistance using the AeroTank Clearomizer with a KangerTech pre-assembled atomizer. A 10-stage micro-orifice uniform deposit impactor (MOUDI) was used to measure the particle size distribution of e-liquids formulations prepared in the laboratory containing nicotine, methamphetamine, and methadone. The MOUDI was well suited to measure particle size, because sticky particles such as PG and VG will adhere to the impaction plates^19^.

## Experimental

This study seeks to understand the relationship between various electronic cigarette modifications and the particle size profile of the aerosols generated by the device using a cascade impactor. The coil resistance, battery voltage, and the composition of the glycols in the e-liquid were varied to determine the impact on particle size formation. E-liquids using 12 mg/mL nicotine were evaluated as well as two e-liquids containing DOTNs, 60 mg/mL methamphetamine and methadone. Methamphetamine is a schedule II CNS stimulant with a history of smoking as a mode of administration. In recent years, arrests have been made with individuals having been found with methamphetamine inside their e-cigarette devices^20^. Methadone, a schedule II synthetic opioid in the same class of compounds as heroin, is primarily taken orally for opioid addiction maintenance. The liquid concentrate formulation of methadone is composed of propylene glycol, which is also a constituent in e-cigarette e-liquids^21^.

### Reagents and supplies

Nicotine, methamphetamine hydrochloride, and methadone hydrochloride were purchased from Sigma Aldrich (St. Louis, MO). The vegetable glycerin and propylene glycol were purchased from Wizard Labs (Altamonte Springs, FL). The Kangertech replaceable atomizers were purchased from Discount Vapers (Oakville, CT), the AeroTank Clearomizer from My Vapor Store (Panama City, FL), and the e-go V v2 variable voltage battery from Vivid Smoke (Irvine, CA). Nicotine, methamphetamine, methadone, nicotine-d4, methamphetamine-d11, and methadone-d9 reference standards were all purchased from Cerilliant Corporations (Round Rock, TX). The methanol and 20 mL scintillation vials were purchased from Fisher Scientific (Pittsburgh, PA). The Micro-Orifice Uniform Deposit Impactor (MOUDI) was purchased by MSP Corporation (Shoreview, MN). The flow meter was purchased from Dwyer (Michigan City, IN).

### Particle size experiments

An AeroTank Clearomizer with a KangerTech pre-assembled atomizer was used in conjunction with an eGo-V2 variable voltage battery to develop a model for research, to easily control variables, and generate the condensation aerosol. This specific device was used due to its popularity in the United States at the time of purchase, and the atomizer was easy to adapt with typical user modifications, such as coil configurations.

Nicotine e-liquid formulations were prepared at 12 mg/mL in 50:50 PG:VG 100% PG, or 100% VG solution in order to measure the impact of PG and VG formulation on particle size. Coil resistance was set at 1.5, 1.8, or 2.2 Ω at 4.3 V. Common battery output voltages were set at 3.9, 4.3, or 4.7 V at 1.8 Ω, the most common resistance for this device. Methamphetamine and methadone e-liquid formulations were prepared at 60 mg/mL in 50:50 PG:VG solution to evaluate the impact of different drugs on particle size and generated with a device operated at the most common battery and resistance settings of 3.9, 4.3, or 4.7 V at 1.8 Ω. E-liquids were stored in a cabinet at room temperature until the experiments were started. The AeroTank clearomizers were filled at half capacity with e-liquid formulation at the day of the experiment and were vortexed prior to aerosol generation. The battery was charged the night prior to the experiments.

A 10-stage micro-orifice uniform deposit impactor (MOUDI), draws the sample through a cascading sequence of nozzles that deposit the aerosol onto plates, was used for particle size analysis^8–10^. Aluminum disks were placed on the plates for stages 1–9 of the MOUDI with filter paper placed on the final stage (stage 10). The mass of the AeroTank Clearomizer containing the e-liquid, each aluminum disk, and the filter were recorded pre- and post aerosolization. The 10-stage MOUDI was operated at a flow rate of 30 L/min. The e-cigarette mouthpiece was positioned flush with the USP induction port (simulated throat) which was connected to the inlet of the impactor to allow sampling of the e-cigarette aerosol. The e-liquids were undiluted. An aerosol was generated 6 times, 10 seconds each, for a single MOUDI collection and performed in triplicate. The particle size distribution data generated by the MOUDI was time-averaged data. Following each experiment, the aluminum disks and the filter were weighed and then were each placed into 20 mL scintillation vials and washed with 1 mL methanol. The USP induction port was washed with 1 mL methanol.

Particle size distributions of the glycols was determined gravimetrically. The change in weight of each aluminum disk and filter was used to determine the total mass of e-liquid collected in the MOUDI. The percent mass recovered on each MOUDI stage was determined for each trial (n = 3) and averaged.

### Analysis of nicotine particle size samples by LC-MS/MS

A previously validated method was used to analyze nicotine concentrations on each stage of the MOUDI using a Quattro micro MS with a Shimadzu LC system (Shimadzu, Kyoto, Japan). Chromatographic separation was achieved on an Agilent Polaris 5-Si A 50 × 3 mm, 5 μm column (Agilent Technologies, Santa Clara, CA). The injection volume was 10 μL with a flow rate of 0.4 mL/min. The total run time for this method was 4.5 minutes and the instrument was operated in multiple reaction monitoring mode (MRM) for the following *m/z* transitions: Nicotine, 163 > 130 and 163 > 117; and nicotine-d4, 167 > 134. A seven-point calibration curve ranging 10–1000 ng/mL of nicotine, along with a blank, double blank control, and nicotine controls were analyzed. Controls were prepared as a limit of quantitation quality control (10 ng/mL), low quality control (30 ng/mL), mid quality control (300 ng/mL), and high quality control (900 ng/mL). The internal standard (100 ng/mL nicotine-d4) was added to each calibrator, blank, control, and samples. Dilutions of the samples were prepared to assure that all samples were bracketed within the calibration range.

### Analysis of methamphetamine and methadone particle size samples by GC/MS

An Agilent 6890 N Gas Chromatograph with a 5973 Mass Selective Detector (MSD) was used for chromatographic separation and detection using a HP-5MS 30 m × 0.25 mm id × 0.25 μm column (Agilent Technologies, Santa Clara, CA) and helium carrier gas.

For methamphetamine, the GC/MS was operated in split mode at 6:1 ratio and a 1 μL injection volume. The helium carrier gas had a flow rate of 35 cm/s and the inlet temperature was set to 275 °C. The GC oven had an initial temperature of 120 °C with a ramp rate of 10 °C/min until 200 °C before undergoing a second temperature ramp of 30 °C/min until 280 °C. The total run time was 10.67 minutes. The MSD was operated in select ion monitoring (SIM) mode with 58, 64, 91, 96, and 134 *m/z* as the selected ions and the quantitation was performed using 58 and 64 *m/z* as the quantitative ions for methamphetamine and methamphetamine-d11 respectively. A six-point calibration curve ranging from 100–2000 ng/mL of methamphetamine, along with a blank, double blank control, and methamphetamine controls were analyzed. Controls were prepared with a limit of quantitation quality control (100 ng/mL), low quality control (150 ng/mL), mid quality control (600 ng/mL), and high quality control (1500 ng/mL). The internal standard (500 ng/mL methamphetamine-d11) was added to each calibrator, blank, control, and samples. Dilutions of the samples were prepared to ensure all samples were bracketed within the calibration range.

For methadone, the GC/MS was operated in split mode at 20:1 ratio and a 1 μL injection volume. The helium carrier gas had a flow rate of 39 cm/s and the inlet temperature was set to 275 °C. The GC oven had an initial temperature of 225 °C with a temperature ramp of 15 °C/min until 285 °C for a total run time of 4 minutes. The MSD was run in SIM mode with the following ions monitored: 72, 223, 294, and 309 *m/z* for methadone and 78, 226, 303, and 318 *m/z* for methadone-d9 and quantitation was performed using 72 and 78 *m/z* as the quantitative ions for methadone and methadone-d9, respectively. A seven-point calibration curve ranging from 100–5000 ng/mL of methadone, along with a blank, double blank control, and methadone controls were analyzed. Controls were prepared with a limit of quantitation quality control (100 ng/mL), a low quality control (150 ng/L), a mid quality control (1000 ng/mL) and a high quality control (4500 ng/mL). The internal standard (500 ng/mL methadone-d9) was added to each calibrator, blank, control, and samples. Dilutions of the samples were prepared to ensure all samples were bracketed within the calibration range.

The geometric mean diameter (GMD), geometric standard deviation (GSD), and mass median diameter (MMAD) were calculated using the methods detailed by Ramachandran and Cooper^22^. Precision was evaluated by calculating each relative standard deviation for each replicate measurement. The threshold for statistical difference was set at a P-value of 0.05 (n = 3).

## Results

### Glycol distribution of nicotine E-liquids aerosol

**Figure 1:**
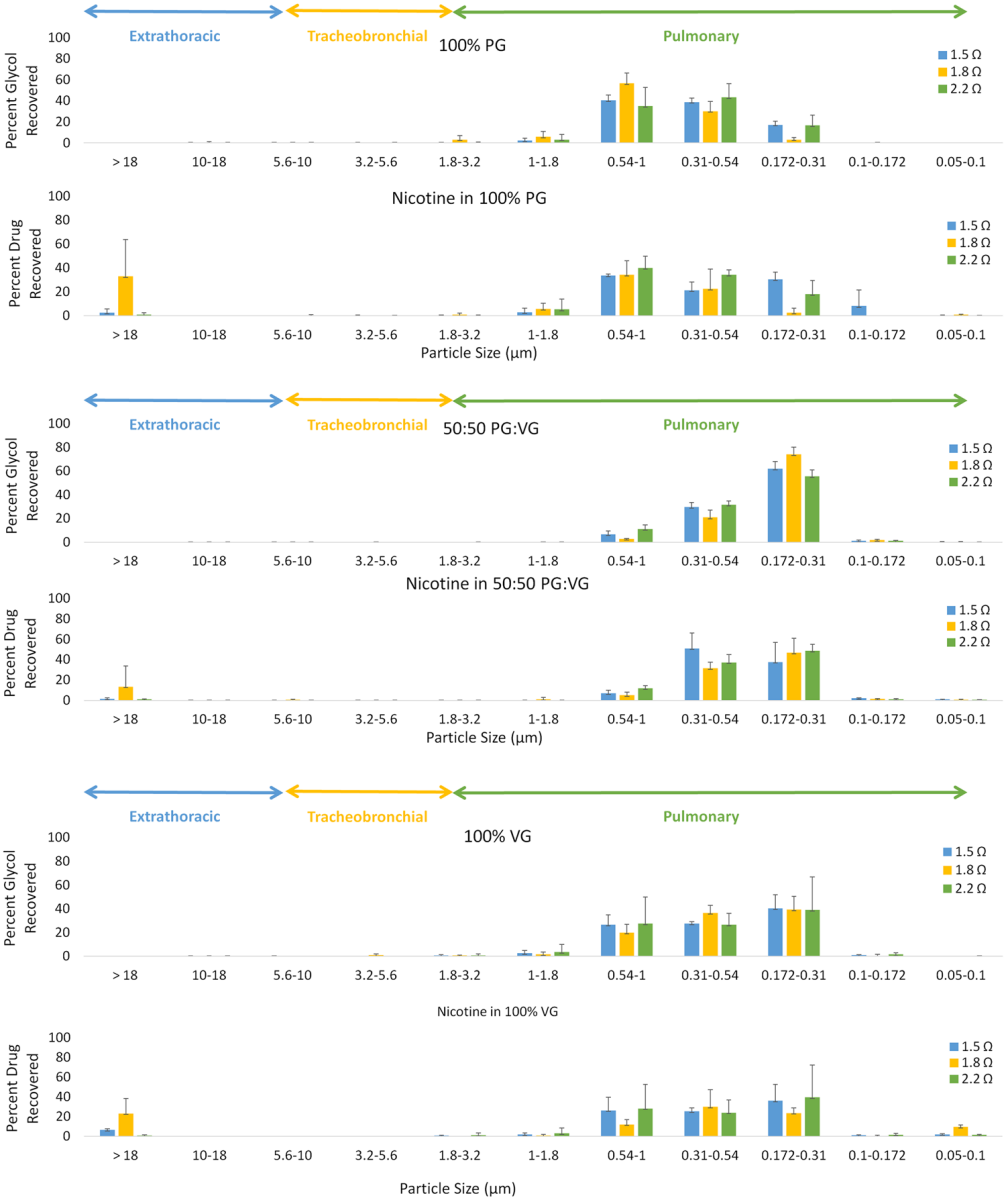
Particle size distribution of glycol and nicotine aerosol by glycol composition and voltage at 1.8 Ω.

**Figure 2:**
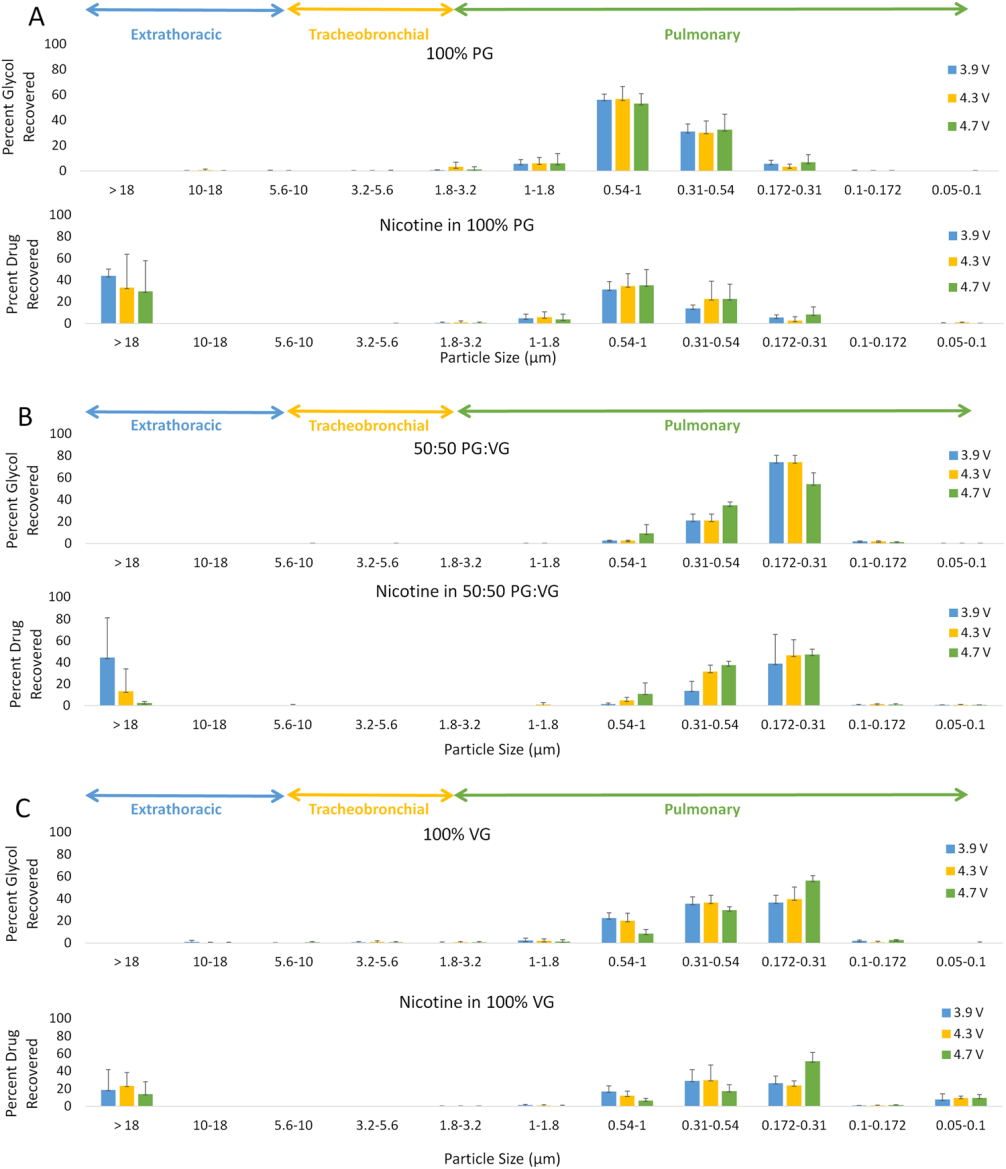
Particle size distribution of glycol and nicotine aerosol by glycol composition and coil resistance at 4.3 V.

The e-liquid with the 100% propylene glycol (PG) composition produced the largest aerosol particles. When varying coil resistance, 90–96% of the 100% PG glycols had aerosol particle sizes between 0.172 μm and 1 μm and the MMAD was 0.55 ± 0.1 µm (Fig. 1). There was no change in particle size distribution as coil resistance was varied (p = 0.193). When varying battery output voltage, 90–93% of the 100% PG glycols had aerosol particle sizes between 0.172 μm and 1 μm and the mean mass diameter (MMAD) was 0.644 ± 0.024 µm (Fig. 2). There was no change in the particle size distribution across the stages or the MMAD when battery output voltage was varied (p = 0.733).

The e-liquid with the 100% vegetable glycerin (VG) composition produced the next largest aerosol particles. When varying coil resistance, 90–96% of the 100% VG glycols had aerosol particle sizes between 0.172 μm and 1 μm and the MMAD was 0.417 ± 0.046 µm. There was no change in particle size distribution across the stages when coil resistance was varied (p = 0.987). When varying battery output voltage, 94–96% of the 100% VG glycols had aerosol particle sizes between 0.172 μm and 1 μm and the MMAD was 0.377 ± 0.026 µm. There was no change in the particle size distribution or the MMAD across the stages when battery output voltage was varied (p = 0.133).

The e-liquid with the 50:50 PG:VG glycol composition produced the smallest aerosol particles. When varying coil resistance, 98% of the glycol aerosol had particle sizes between 0.172 μm and 1 μm and the MMAD was 0.3389 ± 0.009 µm. There was no change in particle size distribution across the stages when coil resistance was varied (p = 0.099). When varying battery output voltage, 97–98% of the glycol aerosol had particle sizes between 0.172 μm and 1 μm and the MMAD was 0.334 ± 0.010 µm. There was no change in the particle size distribution and the MMAD across the stages when battery output voltage was varied (p = 0.199).

### Distribution of nicotine E-liquid aerosol

The nicotine aerosol distribution followed a similar pattern of the glycol distribution of the nicotine e-liquids. When varying coil resistance, 59–93% of the nicotine in the 100% PG e-liquid had particle sizes between 0.172 μm and 1 μm and the MMAD was 0.57 ± 0.12 µm (Fig. 1). There was no change in nicotine aerosol distribution across the stages when coil resistance was varied (p = 0.061). When varying battery output voltage, 59–66% of the nicotine aerosol had particle sizes between 0.172 μm and 1 μm and the MMAD was 0.89 ± 0.32 µm (Fig. 2). There was no change in particle size distribution across the stages and the MMAD when battery output voltage was varied (p = 0.066).

When varying coil resistance, 66–92% of the nicotine in the 100% VG e-liquid had particle sizes between 0.172 μm and 1 μm and the MMAD was 0.455 ± 0.025 µm. There was no change in the nicotine aerosol distribution across the stages and the MMAD when coil resistance was varied (p = 0.837). When varying battery output voltage, 66–75% of the nicotine aerosol had particle sizes between 0.172 μm and 1 μm and the MMAD was 0.451 ± 0.080 µm. There was no change in nicotine aerosol distribution across the stages and the MMAD when battery output voltage was varied (p = 0.430). For the nicotine in the 50:50 PG:VG e-liquid, when varying coil resistance, 83–97% of the aerosol had particle sizes between 0.172 μm and 1 μm and the MMAD was 0.359 ± 0.025 µm. There was no change in the nicotine aerosol distribution across the stages when coil resistance was varied (p = 0.441). When varying battery output voltage, 54–96% of the nicotine aerosol had particle sizes between 0.172 μm and 1 μm and the MMAD was 0.356 ± 0.031 µm. There was no change in the 50:50 PG:VG nicotine e-liquid aerosol when battery output voltage was varied (p = 0.066).

### Distribution of methamphetamine and methadone aerosols

**Figure 3:**
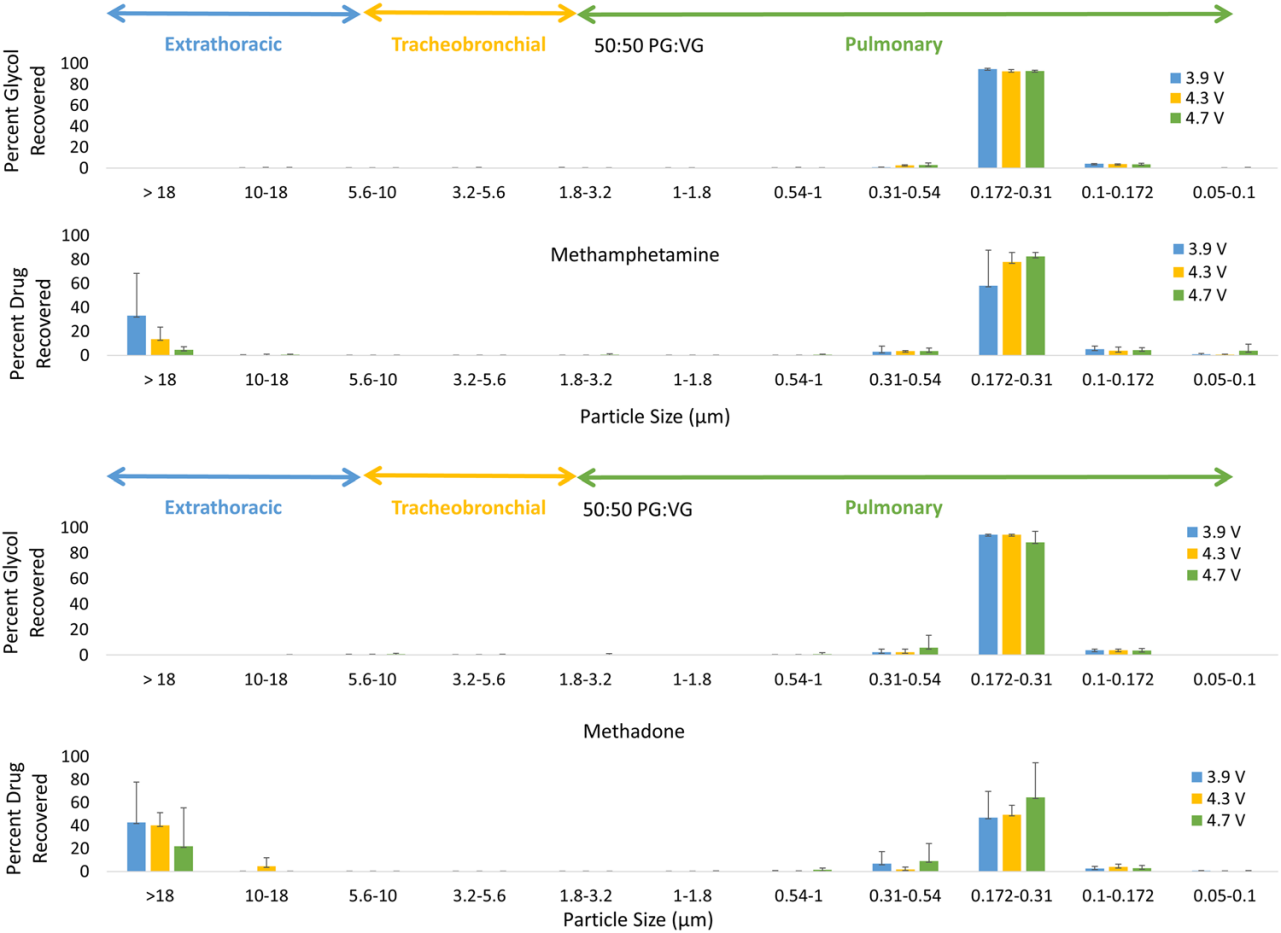
Particle size distribution of glycol, methamphetamine, and methadone by voltage at 1.8 Ω.

The glycols for the methamphetamine and methadone e-liquids had a similar pattern to nicotine glycols in a 50:50 PG:VG e-liquid at 1.8 Ω. For methamphetamine glycols, when varying battery voltage output, 95–96% of the methamphetamine glycols had particle sizes between 0.172 μm and 1 μm and the MMAD was 0.330 ± 0.014 µm (Fig. 3). There was a change in the MMAD of the methamphetamine glycols when the battery voltage output was varied (p = 0.041). For methadone glycols, 95–97% of the aerosol had particle sizes between 0.172 μm and 1 μm and the MMAD was 0.3225 ± 0.0024 µm (Fig. 3). There was no change in the MMAD of the methadone glycols when the battery voltage output was varied (p = 0.057). When comparing against the 12 mg/mL nicotine in a 50:50 PG:VG solution, there was no significant difference between the three drug glycols (p = 0.063).

The methamphetamine and methadone aerosol had a similar pattern to nicotine in a 50:50 PG:VG e-liquid at 1.8 Ω. For methamphetamine aerosol, when varying battery output, 61–86% of the aerosol had particle sizes between 0.172 μm and 1 μm and the MMAD was 0.343 ± 0.016 µm (Fig. 3). There was no change in the methamphetamine aerosol when the battery output voltage was varied (p = 0.494). For methadone aerosols, when varying battery output, 54–75% of the aerosol had particle sizes between 0.172 μm and 1 μm and the MMAD was 0.357 ± 0.056 µm (Fig. 3). There was no change in the methadone aerosol when the battery output voltage was varied (p = 0.223). When comparing against the 12 mg/mL nicotine in a 50:50 PG:VG solution, there was no significant difference between the three e-liquids in terms of drug aerosol (p = 0.864).

## Discussion

Advanced e-cigarette users modify their products by adjusting the coil resistance and battery power in an attempt to optimize their vaping experience and, potentially, deliver more drug in the aerosol. This research group assessed the effect of variable voltage and yield of nicotine per puff in a 50:50 PG:VG solution and the KangerTech atomizer at 1.8 Ω resistance and determined that the change in voltage did have an effect on the yield of nicotine per puff. However, it was concluded that the increased dose per puff with increasing voltage was not practically significant, especially since the puff-to-puff dose exhibited wide variance^23^. Another study measuring particulate matter emissions also effectively demonstrated a significant variation in puff-to-puff aerosol generation in a single device with all variables held constant^24^. A similar phenomenon was observed in this study as the change in battery voltage and coil resistance did not significantly affect the particle size distributions of the three e-liquids. The two variables that had the most effect, yet modest, on the particle size distribution were the glycol composition and the active drug ingredient present in the e-liquid. Despite what appears to be loss of particles >18 µm with increasing battery voltage, the standard deviations in the particle size distributions indicate that e-cigarettes inconsistently generate aerosols such that a significant increase in submicron particles that penetrate into the alveolar region is not demonstrated. Again, it is concluded that the difference in particle size distribution observed between the PG:VG ratios and these 3 drugs is not practically significant in terms of potential drug absorption from the lung tissue into the bloodstream since users modulate their puff topography, intentionally and subconsciously, to control drug effects.

The nicotine containing e-liquid composed of 100% PG produced larger particles in comparison to the 100% VG and 50:50 PG:VG nicotine e-liquids. The larger particle size may contribute to the sensation that e-cig users describe as a “throat hit” in association with e-liquids that have high PG contents^25,26^. Additionally, particle matter emission data demonstrating that 100% PG generated larger total particle concentrations versus 100% VG or mixtures may also support the stronger “throat hit” sensation^24,27^. People who use e-cigarettes for smoking cessation have commented that they prefer e-liquids with a high PG content because of this “throat hit” sensation similar to a traditional tobacco cigarette^26^. In contrast with the 100% PG e-liquid, the 100% VG e-liquid produced aerosol particles that were less than 0.1 μm in size. Aerosol particles that are less than 0.1 μm are referred to as “ultra-fine” particles and are known to result in deep lung deposition^28^. However, with ultra-fine particles, there is a greater chance of the drug being exhaled before it can be absorbed into the bloodstream^29^. To combat this, some e-liquid manufacturers will print instructions on the bottle labels that instruct the e-cig user to inhale and hold the aerosol for a certain amount of time^30^. This method would increase the amount of pulmonary deposition that would lead to more drug absorption into the blood, thereby increasing the bioavailability of the aerosolized drug.

Battery output (V) and resistance (Ω) were found to not have significant impact on particle size distribution, while the ratio of PG to VG did have significant impact on distribution. Other investigators demonstrated that increasing voltage, generally, produced more emissions, however, particle size distribution was not measured^24^. Even so, the higher voltage, or power, was in conjunction with lower resistance, as is typical in dual coil devices like the one was used in the experiment. The aerosol emission is potentially larger because of the increased surface area in the dual coil system which also has double the wick, and not a difference in resistance. Therefore, the findings that higher particle matter emission was a result of higher voltage/power with lower resistance compared to the lower emissions from the higher resistance device in the study is arguable. The finding here-in of larger particle sizes emitted from e-liquids composed of 100% PG contradict other findings that demonstrated PG emits significantly smaller particles than VG, demonstrating the potential impact of varying experimental conditions from one study to another. One study used a cartridge-based cartomizer system with other unknown device settings^31^, another used a dual coil atomizer with, unknown, but fixed airflow^32^, while the other used store bought e-liquids with unverified PG:VG ratios, nicotine concentrations, and flavor constituents^33^. Additionally, one of these studies used a smoking machine, others were directly from the e-cig to instrument; some collected aerosol for 4 seconds, while another for 2 minutes. Variable conditions between studies demonstrating contradictory data and low reproducibility within studies indicate an imperative for standardizing experimental protocols and large-scale studies demonstrating the impact of user modifications such as coil configurations. As example, it is not known if the poly-fill cartomizer compared to an open tank or varying air flow through the atomizer has a significant impact on particle size distribution. As such, comparing the data from a single coil and a dual coil atomizer without knowing other significant variables could be meritless, the same as a tank versus a cartomizer system or, particularly, PG:VG ratios versus un-verified or unknown PG:VG ratios. Czogala and Protano verified that emissions between smoking machines and volunteer ad libitum vaping was significantly different, underscoring the significant impact of variability in puff topography from ad libitum use, in addition to the device variability^24,34^. The conclusion is a complicated array of experiments accounting for all variables to understand the devices and physiological impact is needed.

The nicotine aerosol particles formed by the KangerTech model e-cigarette appear to be similar in size in comparison to the particles formed by traditional cigarettes. Previous studies that analyzed traditional cigarette smoke via impaction or spectral transmission determined that smoke particles range between 0.1–0.5 μm in diameter^33,35–38,40^. This study found that the measurements of these previous tobacco studies are comparable to the aerosol formed by the KangerTech e-cigarette, which formed aerosol particles between 0.3–0.6 μm in diameter. The comparison of various e-cigarette models and brands have reported e-cigarette aerosol particle sizes ranging from 0.1–0.9 μm in diameter as determined by both impaction and spectral transmissions^2,36,37^. These reported particle sizes from e-cigarettes agree with the results obtained in this study. The nicotine aerosol particles followed the same distribution patterns of 100% VG and 50:50 PG:VG, indicating that VG is the driving force for aerosol particle development in this model. The data presented demonstrate wide variability at each of the stages. However, this does not minimize the trending data. Zervas also demonstrated high variability in particle size measurements within the data set and concluded this low repeatability creates conflicts in data from other studies. In conclusion, the particle size data demonstrates that the e-cigarettes are able to produce small particle sizes leading to deep lung tissue deposition and efficient drug delivery, even when glycol composition is altered.

Methamphetamine and methadone exhibited similar particle size distribution behaviors as nicotine. While it was determined that the difference in the MMAD of the three drugs was not statistically significant, the amount of drug recovered on the particle size stage for 0.172–0.31 μm was visually greater for methamphetamine and methadone than nicotine, as demonstrated by the 58–82% and 46–64% and 23–51% drug deposition, respectively, in this range. Methamphetamine smoke has been reported to have less than 1 μm particle size when air in clandestine meth labs was sampled^39^. The methamphetamine aerosol produced in the e-cig system had a MMAD of 0.53 μm in size, indicating that the e-cigarette is capable of creating similar sized drug aerosol particles as when smoked. Heroin, an opioid regularly smoked by users, has a reported MMAD ranges from 2.1–4.1 μm^40^. The MMAD of methadone in this e-cigarette system at 0.64 μm was determined to be similar to the particle sizes of morphine and buprenorphine, which had MMADs between 0.3 and 0.4 μm in size^8–10^. Therefore, both methamphetamine and methadone can be effectively absorbed into the bloodstream when an e-cigarette is used as the vehicle for drug administration. The small particle sizes combined with the user’s modulation of glycol composition and inhalation techniques (i.e. inhale and hold) can improve the bioavailability of methadone and methamphetamine in e-cigarette systems, thereby increasing the potential for overdose.

## Conclusions

The user modifications of glycol composition had some effect on the particle size distribution, but is likely not practically significant, and the coil resistance and battery voltage manipulations had no significant effect. The presence of particles less than 3 μm in size indicates that the drug can reach deep into the lung tissue and deposit into the bloodstream. The presence of particles of this size for methamphetamine and methadone highlight the likelihood of absorption of drugs other than nicotine using electronic cigarettes.
