# Author Correction: The Effect of Electronic Cigarette User Modifications and E-liquid Adulteration on the Particle Size Profile of an Aerosolized Product

**DOI:** 10.1038/s41598-020-62002-1

**Published:** 2020-03-13

**Authors:** Haley A. Mulder, Jesse L. Patterson, Matthew S. Halquist, Leon Kosmider, Joseph B. McGee Turner, Justin L. Poklis, Alphonse Poklis, Michelle R. Peace

**Affiliations:** 1 Department of Forensic Science, Virginia Commonwealth University, Richmond, USA; 2 Department of Pharmaceutics, Virginia Commonwealth University, Richmond, USA; 3 Department of Chemistry, Virginia Commonwealth University, Richmond, USA; 4 Department of Pharmacology & Toxicology, Virginia Commonwealth University, Richmond, USA; 5 Department of Pathology, Virginia Commonwealth University, Richmond, USA

Correction to: *Scientific Reports* 10.1038/s41598-019-46387-2, published online 15 July 2019

This Article contains an error in the Acknowledgements section.

“This project was supported by Award No. NIJ-2016-4305, awarded by the National Institute of Justice, Office of Justice Programs, U.S. Department of Justice and the National Institutes of Health Award No. P30DA033934.”

should read:

“This project was supported by Award No. 2016-DN-BX-0150, awarded by the National Institute of Justice, Office of Justice Programs, U.S. Department of Justice and the National Institutes of Health Award No. P30DA033934.”

